# Inhibition of eCIRP Attenuates Inflammation and Gut Injury After Radiation Combined Injury with Sepsis

**DOI:** 10.3390/medsci14050539

**Published:** 2026-09-01

**Authors:** Fangming Zhang, Gaifeng Ma, Hui Jin, Asha Jacob, Max Brenner, Ping Wang

**Affiliations:** 1 Center for Immunology and Inflammation, Feinstein Institutes for Medical Research, Manhasset, NY 11030, USA; fzhang1@northwell.edu (F.Z.); gma@northwell.edu (G.M.); hjin1@northwell.edu (H.J.); avarghes@northwell.edu (A.J.); mbrenner@northwell.edu (M.B.); 2 Departments of Surgery and Molecular Medicine, Donald and Barbara Zucker School of Medicine at Hofstra/Northwell, Manhasset, NY 11030, USA

**Keywords:** C23, eCIRP, PBI, CLP, systemic and intestinal inflammation

## Abstract

Background: Radiation-induced sepsis resulting from intestinal inflammation and injury is a severe complication of high-dose radiation exposure. High-dose radiation compromises the integrity of the intestinal lining, causing bacterial translocation, systemic inflammation, and sepsis. Extracellular cold-inducible RNA-binding protein (eCIRP), a damage-associated molecular pattern, plays a pivotal role in the pathogenesis of inflammatory diseases and contributes to organ injury. C23, a small molecular peptide antagonist of eCIRP, has demonstrated anti-inflammatory and organ-protective effects during organ-injury indications. However, its role in radiation-induced inflammation and injury is not known. The objective of this study is to evaluate whether C23 mitigates inflammation and injury, leading to improved survival in a murine model of partial-body irradiation (PBI) combined with sepsis. Methods: Mice were exposed to 10 Gy PBI, and at 48 h, they were subjected to cecal ligation and puncture (CLP), a well-established model of sepsis. C23 (8 mg/kg body weight [BW]) or vehicle (saline) was administered subcutaneously at 24 h and 48 h after PBI. Blood and intestinal tissue samples were collected 20 h after CLP (i.e., 68 h post-PBI) for various analyses. In another set of mice after PBI–sepsis, intestinal permeability was also assessed. In an additional cohort of mice subjected to the same experimental procedure, 10-day survival was monitored. Results: Our findings demonstrate that administration of C23 attenuated systemic inflammatory responses, intestinal permeability, intestinal injury, and apoptosis; increased crypt cell proliferation and improved survival after PBI–sepsis. Conclusions: These results reveal a novel protective role of the eCIRP antagonist, C23, in mitigating PBI–sepsis-induced inflammation and injury and represents a promising therapeutic candidate for the treatment of radiation combined injury with sepsis.

## 1. Introduction

Exposure to high levels of radiation can arise from natural disasters, nuclear-facility accidents, or the detonation of nuclear or radiological devices [1,2,3,4,5]. Such exposure can lead to acute radiation syndrome (ARS), a severe illness caused by the rapid absorption of high doses of penetrating radiation [6,7,8]. This may involve total-body irradiation (TBI) or substantial partial-body irradiation (PBI). Radiation injury predominantly affects organs composed of rapidly dividing cells, including the cells in the hematopoietic system (bone marrow), gastrointestinal (GI) tract, and skin. The GI tract, due to the high turnover rate of its stem and epithelial cells, is highly sensitive to radiation-induced DNA damage [9,10]. Consequently, radiation exposure compromises the integrity of the gut’s protective mucus layer and epithelial barrier, facilitating bacterial translocation and disrupting the intestinal microbiome [11,12,13].

Radiation and sepsis are closely interconnected, as acute radiation exposure can lead to the onset of sepsis [14,15]. First, exposure to high doses of ionizing radiation damages living tissues, leading to release of damage-associated molecular patterns (DAMPs) from injured cells, which trigger inflammatory responses. Second, radiation severely compromises the immune system, further promoting systemic inflammation. Third, the gastrointestinal tract plays a central role in initiating this inflammatory cascade, as radiation-induced injury to the intestinal epithelium permits bacterial translocation into the bloodstream and other organs [3,11,12,16]. Radiation-induced epithelial barrier disruption, bacterial translocation, and immune dysfunction contribute to the development of sepsis. As a result, both the morbidity and mortality associated with sepsis are expected to increase following radiation exposure [4,17,18]. Despite this significant clinical concern, no effective treatments currently exist for radiation combined injury with sepsis.

Extracellular cold-inducible RNA-binding protein (eCIRP) has been identified as a potent damage-associated molecular pattern (DAMP) that is released from activated or dying cells [19]. Excessive inflammation driven by eCIRP can lead to cell death and organ damage. The pro-inflammatory effects of eCIRP are associated with its binding to the TLR4/MD2 complex [19]. To identify the region of eCIRP that binds to the TLR4 receptor, we previously designed 32 overlapping 15-mer oligopeptides covering the entire sequence of human CIRP. A series of surface plasmon resonance (SPR) assays were then performed with these peptides, leading to the identification of C23 as a novel eCIRP inhibitor [19]. Subsequently, C23 has been shown to play a significant role in mitigating inflammation and tissue injury under various conditions, including hemorrhage and ischemia–reperfusion injury [20]. However, its role in modulating injury caused by combined radiation and sepsis has not been elucidated. Therefore, we hypothesized that eCIRP released from injured cells directly contributes to inflammation and injury, and that C23 attenuates inflammation, improves gut function, and enhances survival in mice subjected to partial-body irradiation (PBI) combined with sepsis.

## 2. Materials and Methods

### 2.1. Experimental Animals

Healthy C57BL/6 male mice (9–12 weeks old) were purchased from Charles River Laboratories (Wilmington, MA, USA). All mice were housed under a 12 h light/dark cycle, with ad libitum access to food. Animals underwent a minimum 1-week acclimation period prior to experimentation. All experiments were conducted in accordance with the National Institutes of Health Guide for the Care and Use of Laboratory Animals and were approved by the Institutional Animal Care and Use Committee (IACUC) of the Feinstein Institutes for Medical Research (Protocol #24-1093 and 27 February 2025).

### 2.2. Partial Body Irradiation (PBI) in Mice

During irradiation, mice were briefly restrained without anesthesia and placed in fitted containers. Their hind extremities (fibula, tibia, and feet) were shielded with lead tubes, protecting approximately 5% of the bone marrow. The mice were then exposed to PBI at a dose of 10 Gy using an X-ray irradiator (X-RAD 320, PXi, North Branford, CT, USA) at a dose rate of approximately 1 Gy/min, operating at 320 kV and 12.5 mA, with a source-to-surface distance (SSD) of 50 cm. Following irradiation, mice were returned to their cages. Sham-irradiated mice underwent identical handling and placement procedures; however, the X-ray unit remained turned off, and no radiation was delivered.

### 2.3. Murine Model of Polymicrobial Sepsis

At 48 h post-irradiation, cecal ligation and puncture (CLP) was performed to induce polymicrobial sepsis. Mice were anesthetized with 2% inhaled isoflurane and placed in the supine position. CLP was carried out following established protocols [21,22]. Briefly, the abdomen was shaved and disinfected, and a 1.5 cm midline incision was made to expose the cecum. The cecum was ligated with a 5-0 silk suture approximately 1 cm proximal to the distal tip and then punctured once with a 23-gauge needle (or a 22-gauge needle for the survival study). A small amount of fecal content was gently extruded, after which the cecum was returned to the peritoneal cavity. The abdominal wall was closed in two layers using running 4-0 silk suture. We routinely use a 22-gauge needle for the CLP procedure, and significant mortality is observed by 48–72 h. Since this was radiation combined with sepsis and the end point selected was 68 h post-PBI, a slightly smaller needle, i.e., 23-gauge, was used for the CLP procedure. In sham-operated mice, only laparotomy was performed; the cecum was neither ligated nor punctured. Following CLP surgery, animals were resuscitated with 1 mL of normal saline administered subcutaneously and received buprenorphine for analgesia. Mice in the survival cohort additionally received 0.5 mg/kg body weight (BW) imipenem s.c. To prolong survival beyond 48–72 h, one dose of the antibiotic at the time of CLP was administered only in the survival study. After treatment, mice were returned to their cages.

### 2.4. C23 Administration

Prior to PBI, mice were randomly assigned to sham, PBI + CLP and PBI + CLP + C23. C23-treated mice received two subcutaneous doses of C23 (8 mg/kg BW in normal saline): the first dose was administered 24 h after irradiation, and the second one was given immediately following CLP and 48 h after irradiation. The C23 dose was chosen based on our previous studies in sepsis-only condition [23]. Vehicle-treated mice received an equivalent volume of normal saline as administered to the C23-treated group. Sham-operated animals underwent laparotomy without cecal ligation or puncture and served as surgical controls. Following the procedures, mice were returned to their cages. At 20 h after CLP, blood and the small intestine were harvested. For survival study, the mice were monitored daily for 10 days after PBI (8 days after CLP), and the survival rate was recorded.

### 2.5. Tissue Collection

Mice were euthanized via CO_2_ inhalation at 68 h after PBI but 20 h after CLP. The abdominal cavity was then opened, the inferior vena cava (IVC) was punctured for blood collection, and the entire small intestine was harvested. A ~2 cm segment of the small intestine was fixed in 10% formaldehyde for histological analysis. The remaining portion of the small intestine was flash-frozen in liquid nitrogen and stored at −80 °C for subsequent analyses. At the time of tissue harvest, arbitrary numbers were given for each individual mouse and its condition, and those identifications were blinded to the investigator. The decoding was done only after performing statistical analysis; thus, every effort was made to remain unbiased by the investigators.

### 2.6. Intestinal Permeability

Another set of mice (*n* = 8/group) was used for the measurement of intestinal permeability. At 68 h after PBI and 20 h after CLP, mice were anesthetized with 2% isoflurane and underwent oral gavage with 0.5 mL of a 22 mg/mL solution of 4 kDa FITC–dextran (FD4) (CAS No. 60842-46-8, Sigma-Aldrich, St. Louis, MO, USA) in PBS, using a 20-gauge feeding needle. Five hours after gavage, whole blood was drawn by cardiac puncture, and serum was collected by centrifugation at 3000 *g* for 5 min at 4 °C. The fluorescence of FD4 was measured using fluorospectrometry (Synergy H1, BioTek, Winooski, VT, USA), with an excitation wavelength of 485 nm and an emission wavelength of 528 nm. To calculate the fold change of each data point, each experimental value was divided by the mean value of the sham group.

### 2.7. Histological Analysis

Intestinal samples were fixed in formalin, embedded in paraffin, and sectioned into 5 μm slices. Tissue sections were stained with hematoxylin and eosin (H&E), they were examined under light microscopy, and their injury score was evaluated. The scores were based solely on blunting of villi and dilation of villi. Additional tissue sections were deparaffinized; subjected to antigen retrieval with citrate buffer (pH 6.0) in a microwave oven; permeabilized with 0.1% Triton X-100 (MilliporeSigma, Burlington, MA, USA); blocked with 5% normal serum for 1 h; and incubated overnight at 4 °C with a rabbit anti-mouse ZO-1 polyclonal antibody (Catalog No: 61-7300, Thermo Fisher Scientific, Waltham, MA, USA) (1:50 dilution) or rabbit anti-mouse Ki-67 (Catalog No. 280741-AP, Proteintech, Rosemont, IL, USA) (1:500 dilution). Subsequently, the sections were incubated with an HRP-conjugated Horse anti-Rabbit IgG secondary antibody (REF: PK-7200, Vector Laboratories, Inc. Newark, CA, USA) and visualized using 3,3′-diaminobenzidine (DAB; REF: SK-4100, Vector Laboratories). ZO-1 immunostaining was evaluated using ImageJ Fiji software [24] ImageJ 1.54 with Fiji extension package) to obtain a semiquantitative immunoreactive score.

### 2.8. TUNEL Staining

Intestinal tissue sections were stained with TUNEL assay kit (Roche Diagnostics, Indianapolis, IN, USA). Sections were deparaffinized, permeabilized with Triton X-100 (MilliporeSigma, Burlington, MA, USA), and incubated with TUNEL reaction mixture for 60 min at 37 °C in a humidified dark chamber. Upon washing, TUNEL-positive cells were quantified in five randomly selected fields per section with a EVOSM7000 fluorescence microscope (Thermo Fisher Scientific, Waltham, MA, USA).

### 2.9. Enzyme-Linked Immunosorbent Assay (ELISA)

Blood collected from mice that underwent PBI-CLP with and without C23 treatment was spun down at 3000 g for 5 min at 4 °C. Serum levels of TNF-α, IL-6, and IL-1β (both from BD Biosciences, San Jose, CA, USA) were analyzed using ELISA kits, following the manufacturers’ instructions.

### 2.10. Determination of Organ-Injury Markers

Serum levels of AST, LDH, lactate and BUN were measured using specific colorimetric enzymatic assays (Pointe Scientific Inc., Canton, MI, USA), following the manufacturer’s instructions.

### 2.11. Real-Time Quantitative Reverse Transcription–PCR

Total RNA was extracted from intestinal tissue using TRIzol reagent (Invitrogen, Thermo Fisher Scientific, Waltham, MA, USA). Complementary DNA (cDNA) was synthesized using M-MLV reverse transcriptase (Invitrogen, Thermo Fisher Scientific). PCR reactions were performed at a final volume of 25 μL, containing 0.08 μM of each forward and reverse primer, cDNA template, nuclease-free water, and SYBR Green Master Mix (Applied Biosystems, Thermo Fisher Scientific). Amplification and data analysis were conducted using the StepOnePlus Real-Time PCR System (Applied Biosystems, Thermo Fisher Scientific). Mouse β-actin mRNA was used as an internal control, and relative gene expression levels were calculated using the 2^−ΔΔCt^ method. Results are presented as fold change in mRNA expression relative to sham-treated tissues. The forward and reverse primer sequences of mouse genes are IL-6: forward—5′-CCGGAGAGGAGACTTCACAG-3′ and reverse—5′-GGAAATTGGGGTAGGAAGGA-3′; tumor necrosis factor-α (TNF-α): forward—5′-AGACCCTCACACTCAGATCATCTTC-3′ and reverse—5′-TTGCTACGACGTGGGCTACA-3′; MIP-2: forward—5′-CATCCAGAGCTTGAGTGTGA-3′ and reverse—5′-CTTTGGTTCTTCCGTTGAGG-3′; KC: forward—5′-GCTGGGATTCACCTCAAGAA-3′ and reverse 5′-ACAGGTGCCATCAGAGCAGT-3′; β-actin: forward—5′-CGTGAAAAGATGACCCAGATCA-3′ and reverse—5′-TGGTACGACCAGAGGCATACAG-3′.

### 2.12. Western Blotting

Total protein was extracted from intestinal tissue, and concentrations were estimated using Bio Rad protein assay kits (Bio-Rad, Hercules, CA, USA). The protein lysates (50 µg) were separated by SDS-PAGE and transferred to nitrocellulose membranes. After overnight incubation at 4 °C with the primary antibody Claudin-1 (Thermo Fisher Scientific), the membranes were incubated with an HRP-conjugated secondary antibody (LICORbio, Lincoln, NE, USA) for 1 h. The bands were detected with ECL, the membranes were scanned in the Odyssey image system (LI-CORbio, Lincoln, NE, USA), and densitometric analysis was quantified using ImageJ Fiji software.

### 2.13. Statistical Analysis

Sample-size calculation was conducted prior to experimentation. For experiments with the endpoint at 68 h, we estimated sample sizes of 9 animals per group to detect an effect size of 1.5 standard deviations. Sample-size calculations assumed a two-tailed statistical significance of 0.05 or less and a power of 0.8. Our estimated attrition rate was 10%. For the survival study, we estimated a sample size of 20 animals per group based on the expectation that our therapeutic will increase the survival rate from ~40% to ~80%. These estimations were based on both sample-size estimation and our prior experience in conducting similar studies. Data analyses were performed using GraphPad Prism version 8 software (GraphPad Software, San Diego, CA, USA). Data are expressed as mean ± SEM in the figures. One-way ANOVA, followed by the Student–Newman–Keuls (SNK) method, was used for comparisons among multiple groups. Although Tukey’s and Hom-Sidak’s tests are recommended for multiple comparison tests, we have selected the SNK method because it maintains strict alpha control while utilizing a stepwise approach to maximize sensitivity. In fact, we chose SNK within GraphPad Prism because its stepwise distribution logic scales critical ranges to the actual step-distance between ordered means. This allows for a sensitive and realistic stratification of treatment efficacy. Furthermore, because our experimental design features strictly balanced sample sizes across vehicle and treatment, the underlying sampling distribution remains robust against severe error inflation. A *p*-value of ≤0.05 was considered statistically significant.

## 3. Results

### 3.1. C23 Attenuates Systemic Inflammation and Organ Injury After PBI–Sepsis

**Figure 1:**
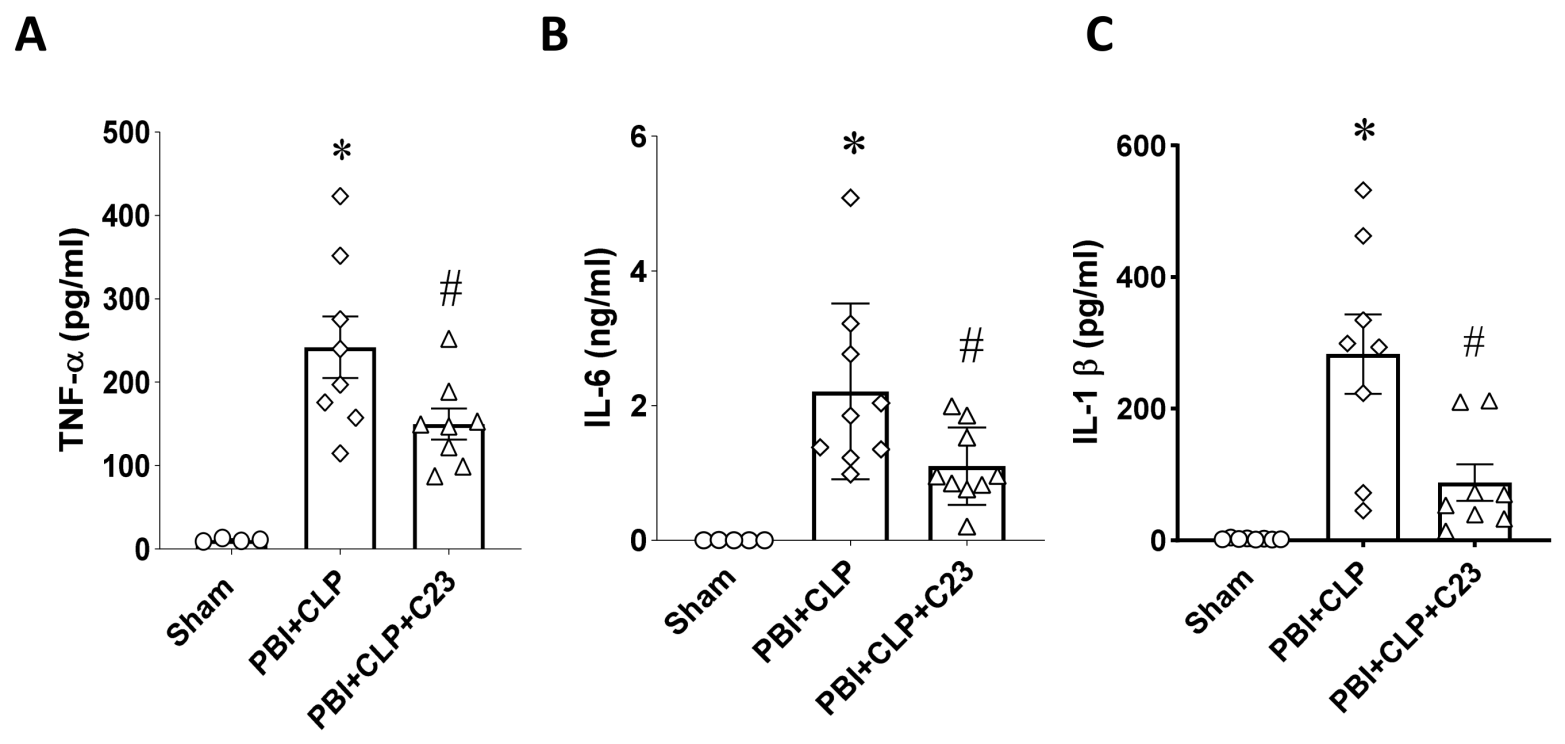
Treatment with C23 attenuates systemic inflammation after PBI–sepsis. C57BL/6 mice were either sham-operated (*n* = 4) or subjected to 10 Gy PBI, followed by CLP with or without two doses of C23 (8 mg/kg BW) [*n* = 8/group] administered at 24 h and 48 h post-irradiation. Plasma samples were collected at 20 h after CLP to measure TNF-α (**A**), IL-6 (**B**), and IL-1β (**C**) using enzyme-linked immunosorbent assay kits. Data are expressed as means ± standard error (SE) of the mean, analyzed by one-way analysis of variance (ANOVA), and compared with Student–Newman–Keuls (SNK) test; * *p* < 0.05 vs. sham; ^#^ *p* < 0.05 vs. PBI + CLP. TNF-α, tumor necrosis factor-alpha; IL-6, Interleukin-6; IL-1β, Interleukin-1β.

**Figure 2:**
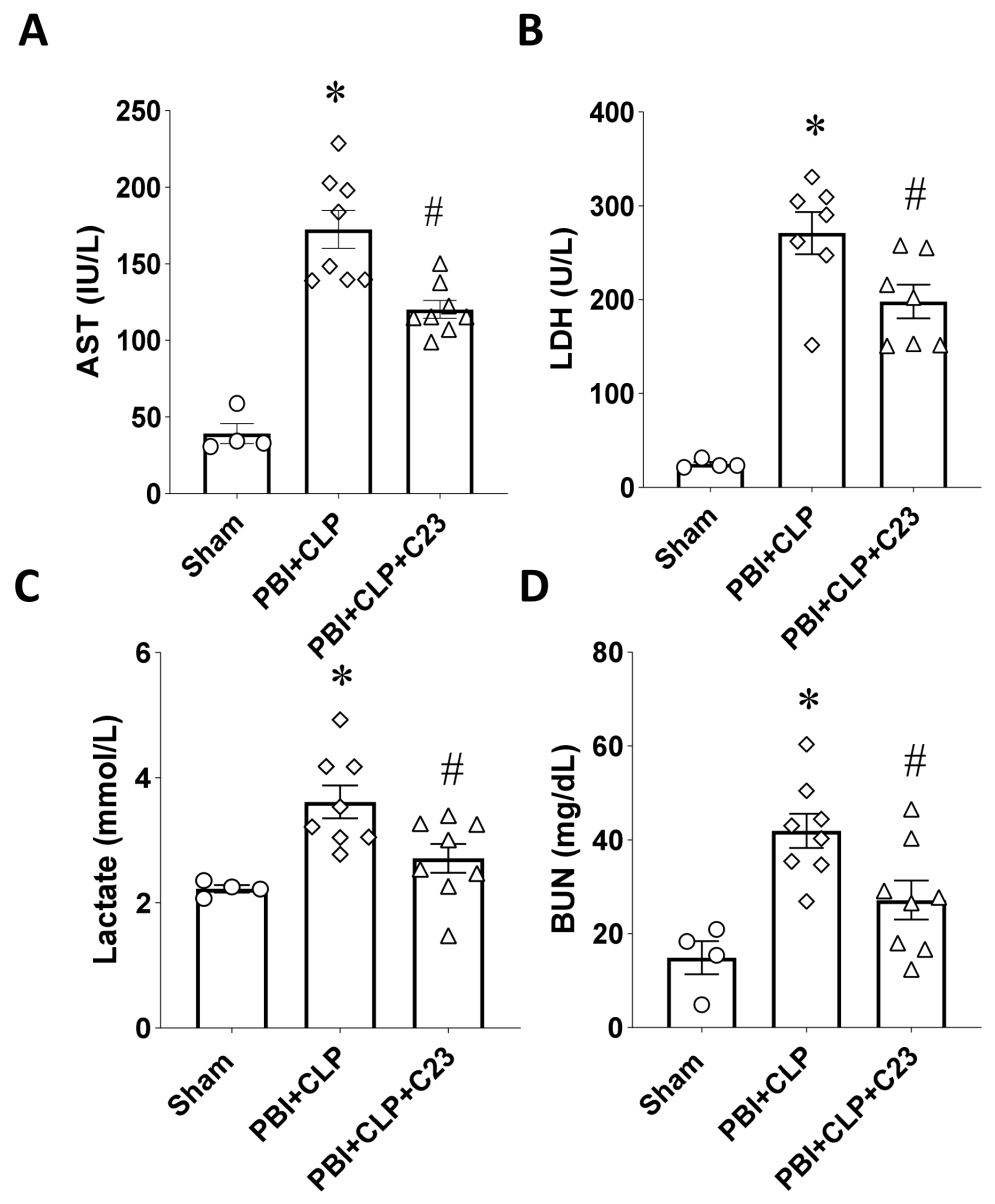
Treatment with C23 reduces organ injury after PBI–sepsis. C57BL/6 mice were either sham-operated (*n* = 4) or subjected to 10 Gy PBI, followed by CLP with or without two doses of C23 (8 mg/kg BW) [*n* = 8/group] administered at 24 h and 48 h post-irradiation. Plasma samples were measured for AST (**A**), LDH (**B**), lactate (**C**), and BUN (**D**) using commercial assay kits. Data are expressed as means ± standard error (SE) of the mean and analyzed by one-way analysis of variance (ANOVA) and compared with Student–Newman–Keuls (SNK) test; * *p* < 0.05 vs. sham; ^#^ *p* < 0.05 vs. PBI + CLP. AST, aspartate aminotransferase; LDH, lactate dehydrogenase; BUN, Blood Urea Nitrogen.

Tumor necrosis factor-alpha (TNF-α), Interleukin-6 (IL-6), and Interleukin-1β are important pro-inflammatory cytokines that serve as key biomarkers of systemic inflammation. To investigate whether C23 attenuates systemic inflammation after PBI–sepsis, blood was collected and analyzed for inflammatory cytokines. Serum levels of TNF-α, IL-6, and IL-1β were dramatically increased in the PBI–sepsis group compared to the sham group. Notably, the administration of C23 significantly reduced the release of TNF-α, IL-6, and IL-1β by 38%, 50%, and 69%, respectively, compared to the vehicle-treated mice (Figure 1A–C). AST, LDH, lactate, and BUN are all markers of organ or tissue injury. To investigate whether C23 can improve organ injury after PBI–sepsis, blood was collected and analyzed for organ-injury indicators. Compared to the sham, plasma levels of AST, LDH, lactate, and BUN were significantly increased in PBI–sepsis. Treatment with C23 significantly decreased these levels by 30%, 25%, 25%, and 35%, respectively, from PBI–sepsis (Figure 2A–D). These data indicate that C23 treatment attenuates systemic inflammation and organ injury after PBI–sepsis.

### 3.2. C23 Reduces Intestinal Inflammation in PBI–Sepsis

**Figure 3:**
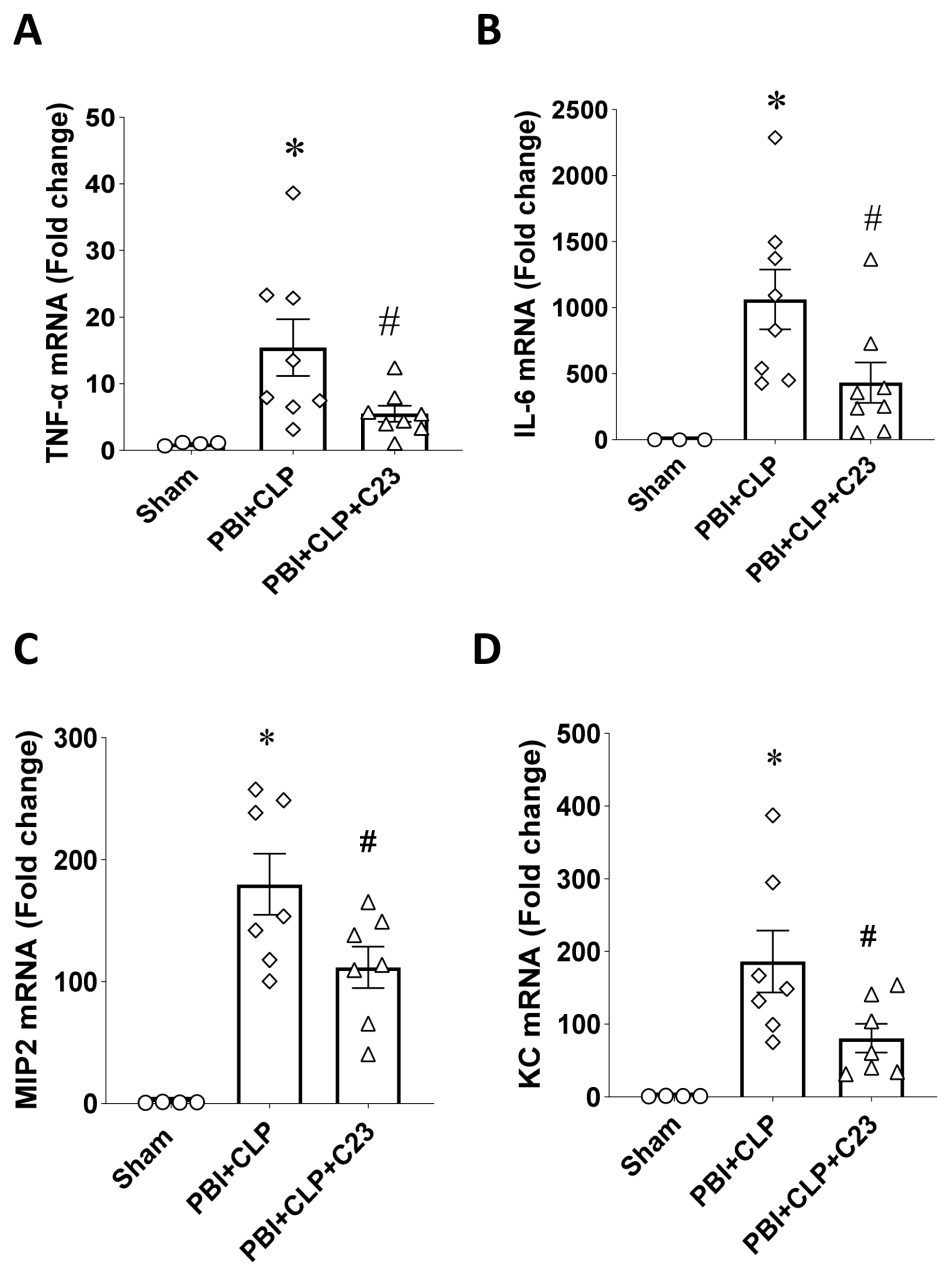
Treatment with C23 suppresses intestinal inflammation after PBI–sepsis. C57BL/6 mice were either sham-operated (*n* = 4) or subjected to 10 Gy PBI, followed by CLP with or without two doses of C23 (8 mg/kg BW) [*n* = 8/group] administered at 24 h and 48 h post-irradiation, and intestinal tissues were harvested. Total RNA was isolated and subjected to real-time PCR to determine mRNA levels of TNF-α (**A**), IL-6 (**B**), MIP-2 (**C**), and KC (**D**). Results are normalized with β-actin as internal control and are expressed as fold induction in comparison to sham. Data are expressed as mean ± SE of the mean (*n* = 4–8 mice/group), analyzed by one-way ANOVA, and compared with SNK test; * *p* < 0.05 vs. sham; ^#^ *p* < 0.05 vs. PBI + CLP.

Radiation causes widespread damage to the intestines by damaging rapidly dividing intestinal cells, and by increasing permeability of the intestinal barrier, leading to sepsis. To further demonstrate the beneficial effect of C23 following PBI–sepsis, intestinal TNF-α and IL-6 mRNA expressions were measured. In the intestine of mice subjected to PBI–sepsis, TNF-α and IL-6 mRNA levels were significantly increased. After C23 treatment, TNF-α and IL-6 mRNA were reduced by 64% and 59%, respectively (Figure 3A,B). Similarly, MIP-2 and KC mRNA expressions in PBI–sepsis mice were significantly increased when compared to sham mice, and importantly, C23-treated mice exhibited a 38% and 57% reduction, respectively, when compared to PBI–sepsis vehicle mice (Figure 3C,D). These data show that C23 treatment attenuates intestinal inflammation after PBI–sepsis.

### 3.3. C23 Attenuates PBI–Sepsis-Induced Intestinal Permeability

**Figure 4:**
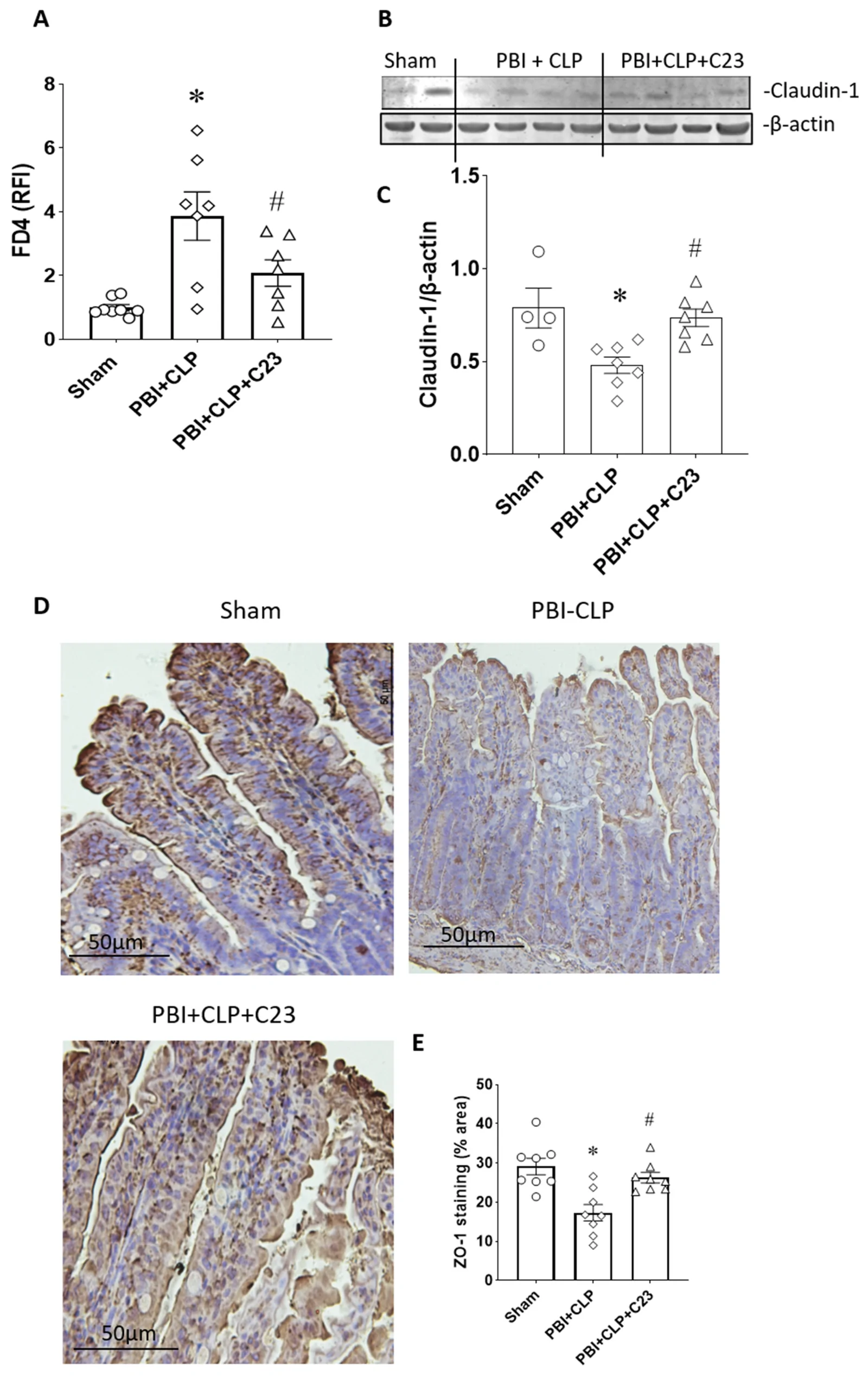
Treatment with C23 decreases PBI–sepsis-induced intestinal permeability. (**A**) At 20 h after PBI–sepsis and treatment, mice that were sham-operated (*n* = 8), PBI + CLP-treated (*n* = 7), and PBI + CLP + C23-treated (*n*= 7) treated underwent oral gavage with FITC–dextran, and 5 h later, blood was collected, and relative fluorescence intensity (RFI) of the FD4 in the plasma was measured to assess intestinal permeability. The values of sham mice were standardized to one for comparison. (**B**) Proteins were extracted and separated by SDS-PAGE and Claudin-1 expression analyzed by Western blotting, and (**C**) densitometric analysis was done with ImageJ Fiji software (**D**). In an additional cohort of mice, 20 h after PBI–sepsis and C23 treatment, intestinal tissues were harvested, and tissue sections were stained with anti-ZO-1 antibody and were examined under light microscopy at 100× original magnifications. Representative images for each group are shown. Scale bar = 50 µm. ZO-1-positive area (brown color) was quantified (**E**) via ImageJ Fiji software. Data are expressed as means ± SE of the mean, analyzed by one-way ANOVA, and compared with SNK test; * *p* < 0.05 vs. sham and ^#^ *p* < 0.05 vs. PBI + CLP.

The barrier function of the intestine is closely related to the development of acute inflammation, and clinical gut-infection patients are usually affected by intestinal-barrier disruption. To determine whether C23 treatment after PBI–sepsis protects the intestinal epithelial barrier, intestinal permeability was assessed using oral gavage of fluorescence-conjugated dyes. After PBI–sepsis, FITC-fluorescence in the serum increased by 3.9-fold compared to the sham, and a 46% decrease in fluorescence was observed in C23-treated mice compared to PBI–sepsis vehicle mice (Figure 4A). In addition, we conducted measurements of Claudin-1, a key transmembrane tight junction protein, by Western blotting to gain insight into the epithelial alterations underlying the observed differences in intestinal permeability. Although the signal was faint, the results showed that Claudin-1 was reduced in PBI–sepsis and the expression was partially restored with C23 treatment (Figure 4B,C). We also conducted measurements of ZO-1, another key tight junction protein, by Western blotting. We did not detect any signal by Western blot due to the large size of the protein, i.e., 220 kDa. Furthermore, we performed immunostaining of the intestinal tissues to assess ZO-1 expression. ZO-1 expression, indicated by brown color staining, in the intestinal villi of PBI–sepsis vehicle mice decreased significantly by 41% compared to sham controls. Remarkably, C23 treatment restored ZO-1 expression to 52% after PBI–sepsis when compared to the vehicle (Figure 4D,E). These data reveal that C23 treatment is associated with recovery of ZO-1 expression and improves intestinal-barrier integrity after PBI–sepsis.

### 3.4. C23 Attenuated Intestinal Injury in PBI–Sepsis

**Figure 5:**
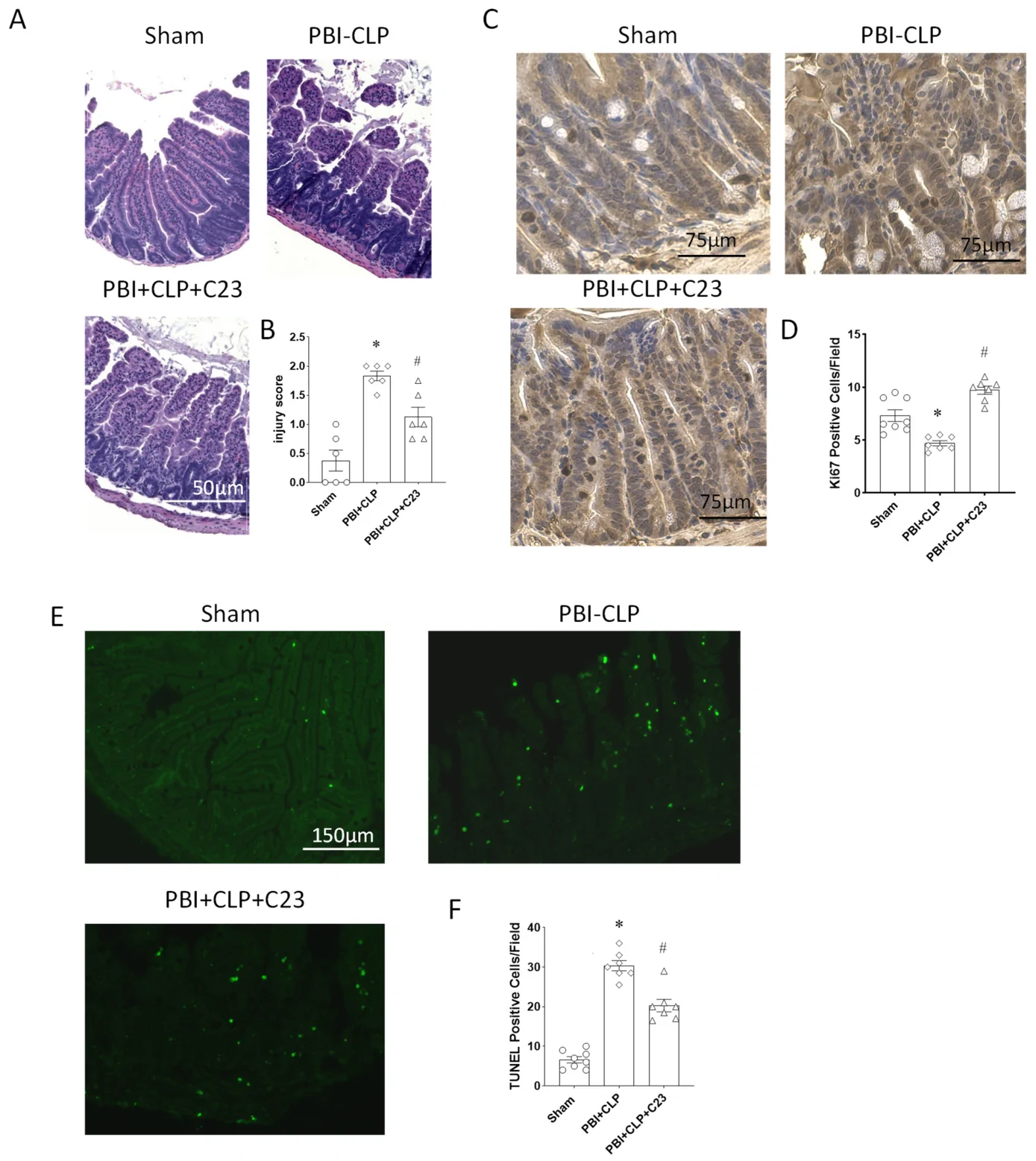
Treatment with C23 improved intestinal integrity and attenuated apoptosis after PBI–sepsis. C57BL/6 mice were either sham-operated or subjected to 10 Gy PBI, followed by CLP with or without two doses of C23 (8 mg/kg BW) [*n* = 6/group] administered at 24 h and 48 h post-irradiation, and intestinal tissues were harvested. Tissues were sectioned, stained with H&E, and examined under light microscopy. (**A**) Representative images of H&E for each group are shown. Scale bar = 50 µm. (**B**) Injury scores of images from (**A**) were evaluated. Tissue sections were stained with Ki67 antibody and examined under 400× magnification. (**C**) Representative images of Ki67 for each group are shown. Scale bar = 75 µm. (**D**) Ki67-positive cells (dark brown spots) per field were quantified. Tissue sections were stained with TUNEL and examined under 200× magnification. (**E**) Representative images of TUNEL staining for each group are shown. Scale bar = 150 µm. (**F**) TUNEL-positive cells (green dots) per field were quantified. Data are expressed as means ± SE of the mean, analyzed by one-way ANOVA, and compared with SNK test; * *p* < 0.05 vs. sham and ^#^ *p* < 0.05 vs. PBI + CLP.

To assess the effect of C23 on intestinal injury after PBI–sepsis, we did H&E staining of the intestinal tissue sections, examined them under the microscope, and scored them (Figure 5A,B). While we did not observe a significant decrease in crypt numbers, PBI + CLP samples exhibited disordered crypts, shortened villi, and dilated villi, which were reversed with C23 treatment. We believe that the moderate appearance of intestinal injury is due to the experimental endpoint being at Day 4.

### 3.5. C23 Increased Crypt Proliferation and Reduced Intestinal Apoptosis in PBI–Sepsis

To determine the effect of C23 on crypt proliferation after PBI–sepsis, we conducted Ki67 staining of the intestinal tissue sections, and cells positive for Ki67 were examined under a fluorescence microscope and calculated (Figure 5C,D). While Ki67 staining decreased in PBI–sepsis compared to control samples, C23 treatment increased the staining slightly beyond sham levels but significantly higher than PBI–sepsis. In addition, we also examined intestinal apoptosis with TUNEL staining of the tissue sections (Figure 5E,F). A 4.6-fold increase in TUNEL-positive cells was seen in PBI–sepsis samples, whereas a significant 33% decrease in TUNEL-positive cells was observed in C23-treated samples compared to PBI–sepsis. These data indicate that C23 increased crypt proliferation while decreasing apoptosis after PBI–sepsis.

### 3.6. C23 Improves Survival Rate in PBI–Sepsis

**Figure 6:**
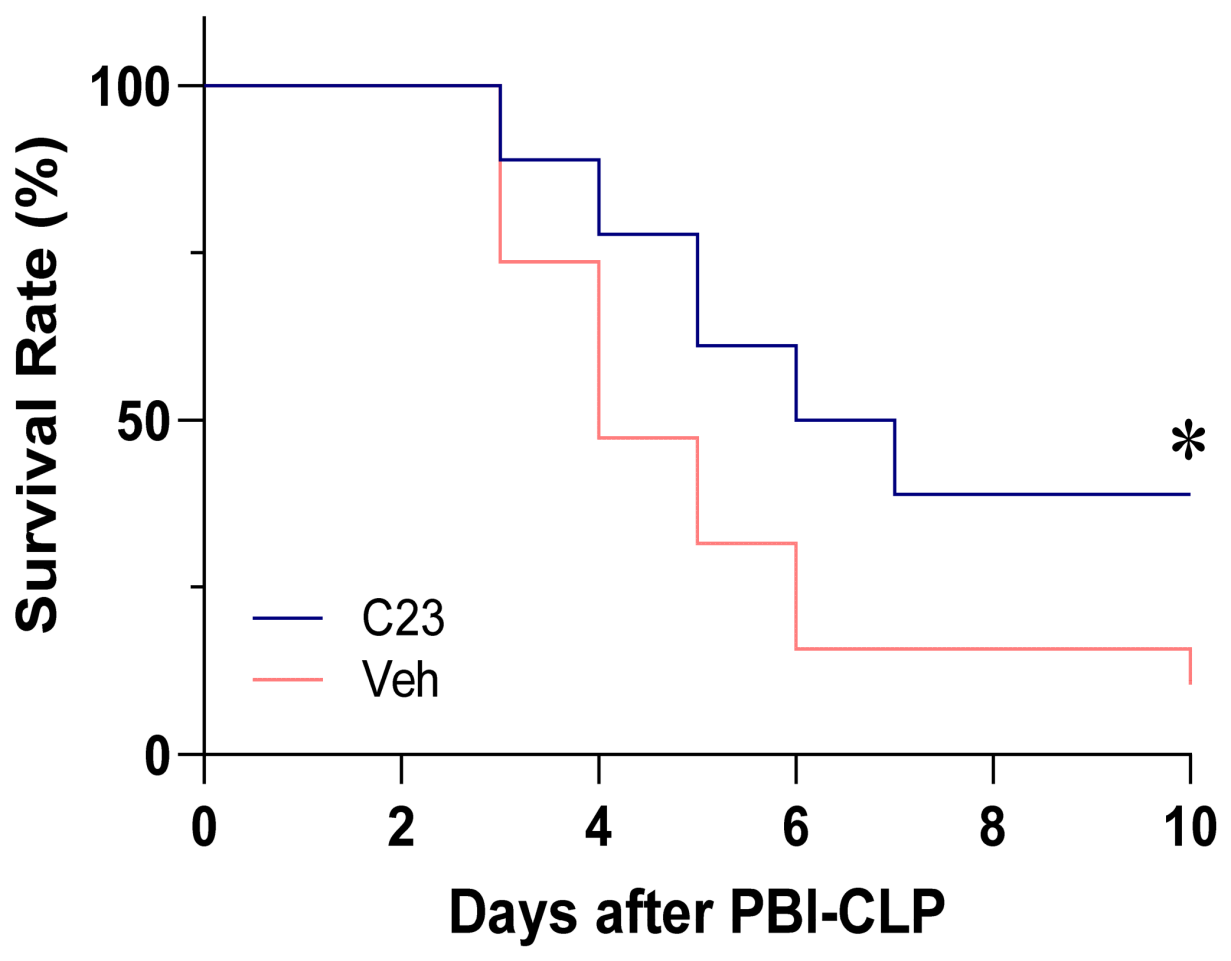
Treatment with C23 improves survival after PBI–sepsis. Mice were subjected to PBI–sepsis and were treated with either vehicle (*n* = 18) or C23 (*n* = 19), and survival was recorded. A 10-day Kaplan–Meier survival curve was generated for the vehicle- and C23-treated groups. * *p* < 0.05 vs. vehicle-treated mice, as determined by the log-rank test.

After demonstrating that C23 reduced organ injury, suppressed inflammation, and improved intestinal integrity in PBI–sepsis mice, we aimed to investigate whether treatment with C23 after PBI–sepsis can provide any survival benefit. The results showed that the PBI-CLP model is a very severe model, resulting in the survival rate in both vehicle and C23 treatment groups being below 50%. However, while vehicle-treated mice exhibited a 10-day survival of 22%, C23-treated mice showed a survival benefit of 47% (Figure 6). These data support that C23 improves survival after PBI–sepsis.

## 4. Discussion

Inflammation is a critical response of normal healthy tissues to ionizing radiation, and it can lead to damage to various organs for a long time after exposure [14]. This inflammation involves damage to the vasculature, migration of leukocytes into the irradiated area, and the release of various immune mediators. These responses strongly depend on the amount of radiation received. As radiation dose increases, there is a greater risk of vascular damage, oxygen deprivation, and cell death. While vascular damage and necrosis initiate inflammation, exposure to high doses of ionizing radiation can lead to other types of cell death, such as apoptosis, which can further trigger inflammation [25]. Severe DNA damage and cell death result in the release of cellular components, like alarmins or DAMPs [26]. In response to ionizing radiation, TLRs play a central role in activating inflammation pathways by interacting with DAMPs. TLRs, through the upregulation of inflammatory mediators such as MAPKs, NF-kB, and COX-2, trigger the secretion of inflammatory cytokines, including TNF-α and IL-6 [25,26]. To study the inflammatory response that occurs after high doses of radiation, we used a model called PBI–sepsis in mice. In our studies, PBI–sepsis was found to increase overall inflammation, as shown by higher levels of proinflammatory cytokines, TNF-α, IL-6, and IL-1β, which were attenuated with C23 treatment. Besides cytokines, systemic markers of organ injury, including AST, LDH, Lactate, and BUN, also increased after PBI–sepsis, and these levels were lowered with C23 treatment. While these data do not specify which specific organs or cell types are protected, they suggest that C23 treatment helps reduce organ damage and systemic inflammation caused by the combined effects of radiation and sepsis.

High-dose radiation causes widespread intestinal damage by targeting rapidly dividing cells, thereby compromising the intestinal permeability barrier and leading to sepsis [27]. In this context, mRNA levels of TNF-α, IL-6, MIP-2, and KC were elevated in the intestine following PBI–sepsis, and these increases were attenuated with C23 treatment. The intestinal inflammation driven by elevated cytokine and chemokine levels further contributes to epithelial permeability barrier dysfunction. In PBI–sepsis, there was an increase in FD4 uptake—a measure of intestinal permeability—and C23 treatment significantly attenuated this effect, suggesting that C23 restored intestinal-barrier integrity.

ZO-1 encoded by the TJP1 gene is a vital scaffolding protein found in epithelial and endothelial cells. These proteins link tight junction components (claudins and occludins) to the actin cytoskeleton [28,29,30]. ZO-1 expression was significantly decreased following PBI–sepsis, and C23 treatment restored its levels. Although the ZO-1 staining appeared relatively uniform, the marked differences in staining intensity between the vehicle and C23 treatment groups suggest that C23 improved intestinal-barrier integrity after PBI–sepsis. Additionally, Claudin-1 expression was reduced in PBI–sepsis and restored with C23 treatment. Histologically, while no significant differences in crypt numbers were observed between PBI–sepsis and C23 treatment samples, PBI–sepsis was associated with disordered crypts, shortened villi, and dilated villi, all of which were reversed with C23 treatment. We believe the moderate degree of intestinal injury observed is attributable to the experimental timepoint of Day 4, which was used for the histological assessment, as well as all other parameters evaluated in this study, with the exception of the 10-day survival curve. Epithelial apoptosis and impairment of crypt cell proliferation are hallmark parameters of radiation-induced intestinal injury. Prior studies have identified gut apoptosis as a therapeutic target in radiation combined injury [31]. We did evaluate intestinal apoptosis with TUNEL staining and crypt cell proliferation using Ki67 immunohistochemistry in the current study. TUNEL staining was increased in PBI–sepsis tissues, whereas the staining was significantly decreased with C23 treatment. While there was a decrease in intestinal apoptosis, it was difficult to assess by TUNEL the specificity of cell types that were affected. In the case of the Ki67 staining, the staining was slightly increased with C23 treatment compared to sham tissues, but it was significantly higher than PBI–sepsis tissues. In this context, we have previously reported that C23 attenuates inflammation and tissue injury in a murine model of intestinal ischemia reperfusion, in which the intestinal injuries observed were similar to those induced by radiation-induced injury [20]. Finally, the PBI–sepsis model employed in this study is highly severe, resulting in a 10-day survival rate of only 22%. Nevertheless, C23 treatment improved the survival rate to 47%, which was significantly higher than that of the vehicle group. Taken together, these data indicate that C23 treatment attenuates intestinal inflammation, intestinal permeability, and apoptosis and increases crypt cell proliferation, ultimately leading to improved survival in PBI–sepsis.

The pathophysiology of radiation combined with sepsis remains largely been unexplored [31,32,33,34]. Ionizing radiation induces DNA damage, and when the damage cannot be resolved through repair mechanisms, it triggers apoptotic cell death signaling pathways, ultimately leading to secondary necrosis. Ionizing radiation affects not only nuclear DNA but also mitochondrial DNA, among other cellular DNA. Since mitochondria serves as the primary generators of ROS within the cells, ionizing radiation-induced mitochondrial damage promotes ROS generation, leading to caspase-9 activation and apoptosis. Radiation-induced cell damage can also alert the immune system to potential danger through the release of DAMPs. Inflammation resulting from excessive DAMP release has the potential to exacerbate radiation-induced tissue and organ damage, underscoring the significance of DAMP modulation as a potential strategy for mitigating radiation combined injury.

The precise mechanism by which C23 confers protection from radiation combined injury has not been completely elucidated. However, our previous study showed that eCIRP induces an inflammatory response in macrophages through the TLR4/MD2 receptor complex, establishing eCIRP is a novel DAMP released under stress conditions [19]. Subsequent surface plasmon resonance (SPR) analysis of oligopeptides derived from eCIRP identified the C23 peptide as having high affinity binding to the TLR4/MD2 receptor complex. Although not directly evaluated in the current study, our prior work has characterized the mechanism of eCIRP-TLR4 signaling and its inhibition by C23. However, since we have not examined downstream signaling molecules (e.g., TLR4 activation, NF-kB, and MAPK) in this study, the inhibition of eCIRP-TLR4 signaling by C23 can only be postulated as the mechanism of action by which C23 reduces inflammation and injury in this PBI–sepsis model of radiation combined injury.

In radiation combined injury, the pathophysiological role of DAMPs remains largely unexplored. However, in severe burns or trauma alone, DAMPs released from the injured tissue have been shown to induce systemic inflammatory response [35,36,37,38,39,40,41,42,43,44]. Similarly, we have demonstrated that eCIRP is a novel DAMP released during sepsis, hemorrhagic shock and ischemia reperfusion injury, and that C23 treatment attenuated injury and inflammation while improving survival in these conditions [19,20]. More recently, we have also shown that high-dose ionizing radiation induces eCIRP release into the peritoneal cavity of irradiated mice and into the cell culture supernatant of irradiated mouse peritoneal cells [45]. Moreover, serum eCIRP levels were increased by more than 2-fold in irradiated mice compared to naïve mice [46]. Although direct evidence of eCIRP inhibition is lacking in the current study, our previous work has demonstrated that circulating eCIRP levels are elevated in both radiation-alone and sepsis-alone conditions and that C23 suppresses eCIRP signaling [19,46]. Thus, targeting DAMPs such as eCIRP may represent a beneficial therapeutic strategy in radiation combined injury.

There are several limitations to our study. First, we did not measure how eCIRP is released during radiation combined with sepsis. Recent studies have unveiled non-apoptotic signaling pathways—such as pyroptosis, necroptosis, and ferroptosis—as additional forms of ionizing radiation-induced cell death [37,47,48,49,50]. Notably, although DAMPs can be actively released through secretory mechanisms, their passive release is primarily linked to cell death processes. Therefore, eCIRP could certainly be released as a consequence of ionizing radiation-induced cell death. Second, the radiation dose was selected based on the findings from our previous PBI study, in which 25% survival was observed at 12.0 Gy over a 30-day period. Since the current study combined radiation with sepsis, we chose a slightly lower dose of 10 Gy. Regarding the treatment dose, the 8 mg/kg regimen was selected based on our previous studies in sepsis-only condition, and C23 was not evaluated in radiation injury alone [23]. As we utilized only a single radiation dose and tested a single treatment regimen, the generalizability of our findings to other radiation exposure scenarios may be limited. Finally, our study used only male mice. Given the well-established sex-dependent differences in physiological responses, immune function and radiation sensitivity, future studies should include female mice to fully explore the therapeutic potential of C23, particularly with respect to dose and time responses. Additionally, because neither a PBI-alone group nor a CLP-alone group was included, it is difficult to determine whether C23 primarily attenuates radiation-induced injury, sepsis-induced injury, or the interaction between the two. We acknowledge the absence of these groups as a further limitation of our study.

## 5. Conclusions

In conclusion, C23 significantly attenuated vascular permeability and improved survival following PBI–sepsis. These findings suggest that targeting DAMPs such as eCIRP could represent a viable therapeutic strategy for radiation injury combined with sepsis. To further develop C23 as a medical countermeasure for PBI–sepsis, additional studies involving optimized timing, dosing, and validation in large animal models are warranted.

## Data Availability

The original data of this study are included in the article. Further inquiries can be directed at the corresponding author.

## Abbreviations

The following abbreviations are used in this manuscript:

eCIRP	Extracellular cold inducible RNA binding protein
PBI	Partial body irradiation
CLP	Cecal ligation and puncture
ARS	Acute radiation syndrome
TBI	Total body irradiation
GI	Gastrointestinal
DAMPs	Danger associated molecular patterns
TLR4	Toll like receptor-4
TREM-1	Triggering receptor expressed on myeloid cells-1
SPR	Surface plasmon resonance
AST	Aspartate aminotransferase
LDH	Lactate dehydrogenase
BUN	Blood Urea Nitrogen
PCR	Polymerase chain reaction
TNF-α	Tumor necrosis factor-alpha
IL-6	Interleukin-6
MIP-2	Macrophage inflammatory protein-2
KC	Keratinocyte-derived cytokine
MAPK	Mitogen activated protein kinase
NF-kB	Nuclear factor-kappa B
COX-2	Cyclooxygenase-2
GI-ARS	Gastrointestinal acute radiation syndrome
HMGB1	High mobility group box 1
IL-1β	Interleukin-1beta
IL-18	Interleukin-18
Gy	Gray
BW	Body weight
FD4	FITC–dextran, 4 kDa
